# Reversible covalent direct thrombin inhibitors

**DOI:** 10.1371/journal.pone.0201377

**Published:** 2018-08-02

**Authors:** Mohanram Sivaraja, Nicola Pozzi, Matthew Rienzo, Kenneth Lin, Timothy P. Shiau, Daniel M. Clemens, Lev Igoudin, Piotr Zalicki, Stephanie S. Chang, M. Angels Estiarte, Kevin M. Short, David C. Williams, Anirban Datta, Enrico Di Cera, David B. Kita

**Affiliations:** 1 Verseon Corporation, Fremont, California, United States of America; 2 Edward A. Doisy Department of Biochemistry and Molecular Biology, Saint Louis University School of Medicine, St. Louis, Missouri, United States of America; University of Parma, ITALY

## Abstract

**Introduction:**

In recent years, the traditional treatments for thrombotic diseases, heparin and warfarin, are increasingly being replaced by novel oral anticoagulants offering convenient dosing regimens, more predictable anticoagulant responses, and less frequent monitoring. However, these drugs can be contraindicated for some patients and, in particular, their bleeding liability remains high.

**Methods:**

We have developed a new class of direct thrombin inhibitors (VE-DTIs) and have utilized kinetics, biochemical, and X-ray structural studies to characterize the mechanism of action and *in vitro* pharmacology of an exemplary compound from this class, Compound 1.

**Results:**

We demonstrate that Compound 1, an exemplary VE-DTI, acts through reversible covalent inhibition. Compound 1 inhibits thrombin by transiently acylating the active site S195 with high potency and significant selectivity over other trypsin-like serine proteases. The compound inhibits the binding of a peptide substrate with both clot-bound and free thrombin with nanomolar potency. Compound 1 is a low micromolar inhibitor of thrombin activity against endogenous substrates such as fibrinogen and a nanomolar inhibitor of the activation of protein C and thrombin-activatable fibrinolysis inhibitor. In the thrombin generation assay, Compound 1 inhibits thrombin generation with low micromolar potency but does not increase the lag time for thrombin formation. In addition, Compound 1 showed weak inhibition of clotting in PT and aPTT assays consistent with its distinctive profile in the thrombin generation assay.

**Conclusion:**

Compound 1, while maintaining strong potency comparable to the current DTIs, has a distinct mechanism of action which produces a differentiating pharmacological profile. Acting through reversible covalent inhibition, these direct thrombin inhibitors could lead to new anticoagulants with better combined efficacy and bleeding profiles.

## Introduction

Anticoagulant drugs have had a profound impact on the treatment of venous and arterial thrombotic diseases, which are a major clinical concern with high prevalence and often fatal outcome [1]. The traditional options for anticoagulation are unfractionated heparin, low molecular weight heparin, and the vitamin K epoxide reductase antagonist (VKA) warfarin. However, all of these treatments suffer from major drawbacks [2–4]. Heparins must be administered parenterally and, due to variations in therapeutic levels, require intensive patient monitoring, making them ill-suited for long-term use. Warfarin, though orally available, has a narrow therapeutic window, large variability in dose response, slow onset and offset of action, and it is contraindicated with many foods and other medications.

Several direct oral anticoagulants (DOACs) have recently been developed to address the significant need, in both short- and long-term therapy, for orally available anticoagulants with rapid onset, reduced bleeding risk, wide therapeutic window, and minimal food or drug interactions [5–7]. These efforts have focused on small-molecule antagonists of two key factors of the coagulation cascade: thrombin (factor II) and activated factor X (FXa).

Work on targeting thrombin has resulted in a single direct thrombin inhibitor (DTI), dabigatran etexilate, which received FDA approval for stroke prevention in atrial fibrillation [8], treatment of deep vein thrombosis (DVT), and pulmonary embolism (PE) [9]. The success of dabigatran demonstrates that effective anticoagulation for venous thromboembolism (VTE) prevention and stroke prophylaxis can be achieved using an oral, small-molecule inhibitor of thrombin.

Efforts focused on targeting FXa have led to the development of rivaroxaban, apixaban, and edoxaban [7]. These drugs are all oral, small-molecule inhibitors that have been approved for use in North America and Europe.

Compared with the VKAs, the new generation of DOACs have more predictable anticoagulant response and are effective in the prevention and treatment of venous thromboembolism, stroke, and systemic embolism in patients with nonvalvular atrial fibrillation. Despite the advantages of the DOACs over the VKAs, their use is limited or contraindicated under some circumstances and the bleeding liability remains high [10–13]. Hence, there is still an unmet medical need for anticoagulants with comparable *in vivo* pharmacological efficacy and superior safety properties.

A retrospective analysis of successful drug launches has revealed that approximately 30% of all small-molecule pharmaceuticals act by covalent modification of the target [14,15]. Representative examples include blockbuster agents such as the platelet antagonist clopidogrel (Plavix) and the proton pump inhibitor lansoprazole (Prevacid). Targeted covalent inhibition has a number of potential advantages, including prolonged pharmacodynamic exposure with more complete inhibition, leading to less frequent or lower dosing. Furthermore, the potential risks of covalent modification, e.g., the risk of forming immunogenic adducts, can be minimized by selective and transient modification [16]. Another potential advantage associated with the nonequilibrium binding of covalent inhibitors is their limited competition with high endogenous ligand concentrations. Unlike the equilibrium competitive inhibitors, covalent inhibitors may therefore allow the desired efficacy to be achieved at lower drug concentrations [16].

We have developed a class of direct thrombin inhibitors (VE-DTIs). Here, we describe the mechanism of action of Compound 1, an exemplary VE-DTI, which acylates the active site S195 of thrombin in a transient manner (note that throughout this paper, amino acids are numbered according to the classical chymotrypsin numbering), thereby utilizing the advantages of covalent reversible inhibition. We illustrate how this mechanism may lead to novel pharmacology.

## Materials and methods

### Enzymes and reagents

Enzymes were purchased commercially from Haemtech Biopharma Services, except for those used in the experiments shown in section “Binding of dansyl Compound 2 to thrombin” below, where wild-type thrombin and mutant S195A were expressed as prethrombin-2 in *E*. *coli*, refolded, and purified to homogeneity as previously described [17]. Chromogenic substrates S-2238, S-2302, S-2366, and S-2765 were purchased from Diapharma, Spectrozyme was purchased from Sekisui Diagnostics, and fibrinogen was purchased from Sigma.

### Enzyme inhibition assays

IC_50_s were determined by first preincubating the enzyme with the compound in assay buffer (5% DMSO final concentration) for 10 min. Then, substrate was added, and the enzyme activity was measured based on the increase in absorbance at 405 nm after 10 min using either a Cytation3 or Synergy H1 plate reader (Biotek). Final reaction volumes were 100 μL.

The assay buffer used for thrombin (3 nM final concentration), FXa (5 nM final concentration), and plasmin (10 nM final concentration) was 50 mM Tris (pH 7.4), 100 mM NaCl, and 0.2% PEG 8000. For FXIIa, the buffer also contained 50 mM imidazole. For trypsin (3 nM final concentration), aPC (25 nM final concentration), FVIIa (12 nM final concentration, complexed with 25 nM rh-tissue factor), and FXIa (10 nM final concentration), 20 mM CaCl_2_ was included in the buffer. The buffer for FIXa (50 nM final concentration) was 50 mM Tris, 100 mM NaCl, 5 mM CaCl_2_, and 30% ethylene glycol.

The chromogenic substrates and final concentrations used in each reaction were S-2238 (125 μM) for thrombin and (250 μM) for FVIIa; S-2302 (250 μM) for FXIIa; S-2366 (250 μM) for aPC and (250 μM) for FXIa; S-2765 (200 μM) for FXa and (125 μM) for trypsin; S-2251 (125 μM) for plasmin; and Spectrozyme (1 mM) for FIXa.

Enzyme activity with clot-bound thrombin was monitored using the substrate S-2238. Fibrin clots were generated by incubating a 3 mg/mL fibrinogen solution with 20 nM thrombin in assay buffer (20 mM HEPES (pH 7.4), 150 mM NaCl, 10 mM CaCl_2_) for 30 min at 37°C in a 96-well microtiter plate (100 μL final volume). The clots were then extensively washed in the microtiter plate and resuspended in thrombin assay buffer, see above. The absence of free thrombin was confirmed by assaying an aliquot of the supernatant for thrombin activity. Various dilutions of Compound 1 in DMSO were then added to different wells of the microtiter plate and incubated for 10 min at RT, followed by addition of a solution of S-2238 (final concentration 250 μM). After an additional 10 min, an aliquot of the supernatant was transferred to a new clear well plate and the absorbance at 405 nm was read immediately. For comparison, the inhibition of thrombin in solution was run in parallel as described above.

### Product formation after incubation of Compound 1 with thrombin

Thrombin at 5 μM was incubated with Compound 1 at 1 μM for 20 min, or at 10 μM overnight, in buffer containing 50 mM Tris (pH 7.4), 100 mM NaCl, and 0.2% PEG 8000. The reaction was then quenched with acetonitrile and centrifuged at 4°C, 1000x RCF, for 10 min to pellet the protein. The supernatant was then analyzed by LC-MS/MS for Compound 1, Compound 3, and 2-methoxybenzoic acid. Quantification of the compounds was performed by LC-MS using a Shimadzu Nexera X2 UPLC system coupled with a Sciex QTrap 5500. Chromatography was performed on a LunaOmega Polar C18 analytical column (1.6 μm, 50 x 2.1 mm). The mobile phases were 0.025% formic acid in water and 0.025% formic acid in acetonitrile.

### Preincubation kinetics experiments

Various concentrations of compound were mixed with human α-thrombin for different durations, after which a large excess of substrate S-2238 was added (approximately 50 x *K*_*M*_) to outcompete further inhibition. The remaining enzyme activity after each preincubation was then monitored. The point *t* = 0 was defined as the activity without preincubation (i.e., thrombin added to a premixed solution of compound and S-2238).

Each well in three rows of a 96-well microtiter plate were filled with assay buffer (50 mM Tris, pH 7.4, 100 mM NaCl, 0.2% PEG 8000), followed by a solution of DMSO with various concentrations of Compound 1. For the *t* = 0 row, S-2238 in assay buffer was added (total of 100 μL per well). In each of the other two rows, thrombin was added and allowed to react for 6 sec and 22 sec, respectively, before the addition of the substrate. Finally, thrombin was added to the *t* = 0 row, and the absorbance at 405 nm was immediately read in kinetic mode for 5 min. Final concentrations were 250 μM S-2238, 10 nM thrombin, and 5% DMSO. Reactions were run at 25°C and 1 atm.

The data were analyzed as described in Doucet et al. [18] and Parsons et al. [19] using the simplified model for the inactivation of a serine protease with an acylating agent

$$E+I\begin{array}{c} \overset{k_{1}}{\to }\\ \underset{k_{2}}{\leftarrow } \end{array}E*I\overset{k_{inact}}{\to }E-I^{\prime }\overset{k_{3}}{\to }E+P,$$ (1)

with E*I being the noncovalent Michaelis complex, E-I’ the covalently inactivated enzyme, and P the hydrolysis product resulting from deacylation of the enzyme. The deacylation rate, *k*_3_, as measured by the spontaneous recovery of enzyme activity is very slow (*t*_1/2_ ~ 8 h) and was neglected for this analysis.

The enzyme activity remaining after incubation with each inhibitor concentration was then determined as a percentage of the activity observed in the absence of inhibitor. To correct for inhibition occurring between substrate addition and data collection, these values were then normalized to the *t* = 0 value for each inhibitor concentration (the no-preincubation condition of the first row). The observed inactivation rate, *k*_obs_, was determined as the slope in a semilogarithmic plot of activity versus time

$$\ln\frac{\left[E](t\right)}{{\left[E\right]}_{0}}=-k_{obs}t.$$ (2)

To determine the rate constant for acylation, *k*_inact_, and the apparent inhibition constant, *K*_i_, the values for *k*_obs_ were fitted to the hyperbolic equation following Michaelis-Menten kinetics

$$k_{obs}({\left[I\right]}_{0})=k_{inact}*\frac{{\left[I\right]}_{0}}{K_{i}+{\left[I\right]}_{0}}.$$ (3)

Statistical analysis was performed using GraphPad Prism 7.02. For the experiments presented in this paper, *n* = 2 and the mean and standard deviation are reported for each parameter.

### Progress curve kinetics experiments

Enzyme was added to a solution of chromogenic substrate and inhibitor in assay buffer (total of 100 μL per well), and absorbance at 405 nm was read immediately in kinetic mode for up to 30 min. Activity was corrected for background (wells with no enzyme) and time *t* = 0 was synchronized using the initial linear phase of the signal from wells with no inhibitor. The data for the initial time points, up to at least 65% reaction progress, were analyzed as described in Wakselman et al. [20]. The absorbance at 405 nm was fitted to a one-phase exponential association model

$$A_{405}(t)=A{(1-e}^{-k_{obs}t}),$$ (4)

where *k*_obs_ = *k*_obs_([I]’_0_) is a function of the inhibitor concentration, given by Eq (3).

Due to the presence of substrate in the inhibition reaction, the effective inhibitor concentration, [I]’, is diminished relative to the total concentration, [I], by a factor 1 –*α*, where *α* is equal to the fraction of enzyme in the mixture occupied by the substrate

$$\alpha =\frac{{\left[S\right]}_{0}}{{\left[S\right]}_{0}+K_{M}}.$$ (5)

Thus, when fitting to Eq (3), the extracted values *k*_*obs*_ for each well were plotted against the modified inhibitor concentration

$${[I]\prime }_{0}=(1-\alpha ){\left[I\right]}_{0}.$$ (6)

The final reaction condition for thrombin was 20 nM enzyme and 50 μM S-2238; for the other enzymes, the concentrations were identical to those used for the IC_50_ determination. All reactions were run at 25°C and 1 atm. Statistical analysis was performed using GraphPad Prism 7.02. For the experiments presented in this paper, *n* = 2 and the mean and standard deviation are reported for each parameter.

### Recovery of thrombin activity

Thrombin (10 nM) was incubated with an excess of Compound 1 (200 nM) for 1 h, which resulted in complete inhibition of thrombin. Excess inhibitor was then rapidly removed by buffer exchange using Micron-10 Centrifugal filters (Merck Millipore). Aliquots of the retentate were then assayed for thrombin activity using the peptide substrate S-2238 (125 μM final) and compared to untreated enzyme undergoing the same experimental procedure.

### Binding of a dansyl probe to thrombin

**Fig 1:**
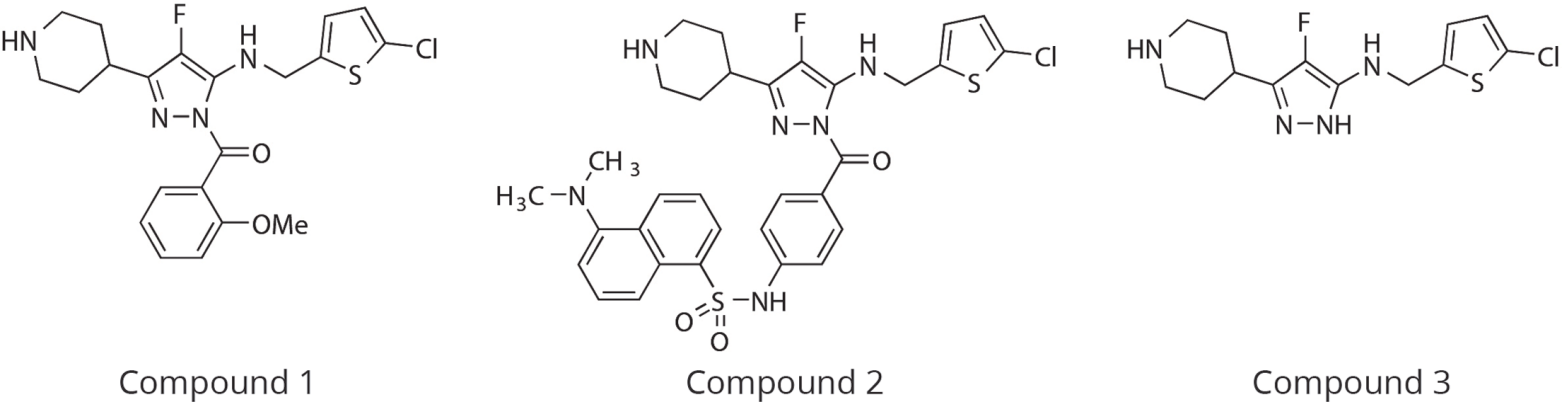
Structures of Compound 1, the dansylated Compound 2, and the deacylated Compound 3 described in this paper.

Dansyl-labeled Compound 1, referred to as Compound 2, is shown in Fig 1, middle. Compound 2 at 1 μM was incubated with WT human α-thrombin (80 nM) or the active site mutant protein S195A at room temperature for 10 min. Then, fluorescence measurements (excitation 280 nm, emission 340 nm) were recorded in kinetic mode.

### SDS-PAGE analysis of Compound 2 binding to thrombin

Compound 2 at 50 μM was incubated with thrombin (6.5 μM) at room temperature for 30 min. The sample was then treated with a hot 0.1% solution of SDS to denature the enzyme and release any noncovalently bound molecules. The samples were then analyzed by gel electrophoresis in the presence of SDS and illuminated with UV light for detection.

### Protein crystallization

For complex formation, 2 mg human α-thrombin (7.1 mg/mL in 50% v/v glycerol) were mixed with 0.5 mM Compound 1 in DMSO and dialyzed overnight into a solution of 20 mM sodium citrate and 1.3 mM Na_2_SO_4_ (pH 5.8). After dialysis, another 0.5 mM compound were added and left to incubate at room temperature for 1 h. The protein was centrifuged for 5 min at 4°C and 8000x RCF. The pellet, including about 120 μL of buffer, was mixed with 100 μL of a solution containing 50 mM sodium phosphate (pH 7.3), 190 mM sodium chloride, and 0.2 mM Compound 1. The crystal used for data collection was grown from the JCSG+ screen in well A10. The crystal was grown at 20°C using a MRC 3-well plate and in a 150 + 150 nL drop with a reservoir of 0.2 M potassium formate and 20% w/v PEG 3350. Five days after crystallization setup, thin, plate-like crystals with dimensions up to about 0.1 x 0.1 mm were observed, collected, and stored at -173°C.

### X-ray data collection, structure determination, and refinement

Data were collected at 100 K at station I03 at the Diamond Light Source, Didcot, England (λ = 0.97625 Å). Data were processed using the XDS [21] and Aimless programs [22]. Initially, the data were processed with a traditional spherical resolution cutoff, later with an ellipsoidal resolution cutoff. The structure was determined using Phaser [23] and the PDB entry 1ABJ as a model for molecular replacement. Two thrombin molecules were found in the asymmetric unit. The structure was refined in Refmac [24] and Buster [25]. Throughout refinement, NCS restraints were applied to simplify convergence. Model building was done in Coot [26]. The initial model was refined using NCS restraints in Refmac to 2.89 Å resolution with R_model_/R_free_ = 0.23/0.29. This model was used for molecular replacement in Buster after reprocessing of all images in Autoproc [27], using an anisotropic data cutoff as determined by the program STARANISO [28]. The final refinement in Buster was made with data between 30 and 2.9 Å, but the data was cut irregularly between 2.9, 4.5, and 4.3 Å in different directions, see Table 1 for parameters.

**Table 1:** X-ray data collection and refinement statistics.

	Final refinement in Buster after anisotropic removal of weak data (STARANISO processing)
**Resolution**	30–2.90 (3.18–2.90) Å
**Wavelength**	0.97625 Å
**Space group**	C222_1_
**Unit cell**	a = 91.7 Å, b = 99.7 Å, c = 146.0 Å
**Completeness**	72.1% (36.0%)
**Redundancy**	6.5 (6.2) ^a^
**No. of observations**	70 885 (1400) ^a^
**No. unique reflections**	10 936 (1287)
**<I/σ(I)>**	8.2 (1.6) ^a^
**CC(1/2)**	99.3% (50.4%) ^a^
**R**_**merge**_ **(I)**	19.2% (119.8%) ^a^
**R**_**cryst**_ **(F)**	17.6% (20.4%)
**R**_**free**_ **(F)**	25.4% (31.4%)
**No. of non-hydrogen atoms**	4614
**No. of water molecules**	22
**r.m.s. deviations from ideal** **geometry: Bond lengths**	0.010 Å
**Bond angles**	1.2 deg
**Mean B-factor, protein chains**	54.5 Å^2^, 62.2 Å^2^, 84.6 Å^2^, 80.5 Å^2^
**Mean B-factor, ligands**	88.2 Å^2^, 95.4 Å^2^
**Mean B-factor, water**	28.6 Å^2^

^a^ Outer resolution shell (2.95–2.90 Å).

### Fibrinogen cleavage

The assay was performed in a 96-well microtiter plate with a mix of human platelet-poor plasma (20% final concentration) in buffer containing 20 mM HEPES (pH 7.4), 150 mM NaCl, and the peptide GPRP (1 mM final concentration; to prevent clot formation). Following addition of the compound in DMSO, human α-thrombin (10 nM final concentration) was added and incubated at room temperature for 30 min. The reaction was quenched with 5 μL of perchloric acid (70% in water) and centrifuged at 4°C, 1000x RCF, for 10 min to pellet the proteins. The supernatant was then analyzed for quantitation of fibrinopeptide A by HPLC using the Aeris 3.6 μm peptide XB-C18 column using 0.1% trifluoroacetic acid and acetonitrile as the mobile phases.

### Protein C activation and thrombin-activatable fibrinolysis inhibitor (TAFI) activation by thrombin

The assays are similar to the method described in Hall et al. [29]. A thrombin/thrombomodulin complex was first created by premixing α-thrombin (30 nM) with thrombomodulin (300 nM) in buffer containing 50 mM Tris (pH 7.4), 150 mM NaCl, 0.2% PEG, and 2.5 mM CaCl_2_. This complex (3 nM final thrombin concentration) was then incubated with compound in DMSO at room temperature for 10 min. Then, protein C or TAFI was added (200 nM final concentration) and incubated at 37°C for 20 min. Subsequently, thrombin activity was completely inhibited by adding PPACK (0.2 μM final concentration). Then, the activated protein C substrate S-2366 (400 μM final concentration) or the TAFI substrate FA-AR (3-(2-furyl)acryloyl-Ala-Arg-OH; 400 μM final concentration) was added and the absorbance at 405 nm or 336 nm, respectively, was recorded after incubation for 60 min at room temperature or 37°C for protein C and TAFI, respectively.

### Thrombin generation assay (TGA)

The assay was performed with human platelet-poor plasma (70% final concentration) in buffer containing 20 mM HEPES (pH 7.4), 150 mM NaCl, and compound in DMSO (4% final concentration). The assay also contained phospholipids (approx. 3 μM) and Dade Innovin (1/5000 final dilution of stock). The reaction was triggered by the addition of a solution containing substrate (Z-GGR-AMC, 0.5 mM final concentration) and CaCl_2_ (15 mM final concentration). The reaction was then monitored kinetically by fluorescence (excitation 380 nm, emission 460 nm) at 37°C for 90 min.

### Prothrombin time (PT) and activated partial thromboplastin time (aPTT) clotting assays

Both PT and aPTT assays were performed as recommended by the vendor (Diagnostica Stago). Briefly, citrated normal pooled human platelet-poor plasma was mixed with test compound (2% DMSO) at 37°C. Subsequently, either the aPTT assay reagent or PT assay reagent was added and mixed thoroughly. CaCl_2_ was then added to trigger the clotting process. The time for clot formation was measured in seconds and the test compound concentration to double the clotting time (CT_2x_) was determined.

### Plasma stability assay

The compound at 20 μM (2% final DMSO concentration) was incubated with CD-1 mouse (Innovative Research), SD rat (Innovative research), or human plasma (George King BioMed) at 37°C for up to three hours. Aliquots were taken out at different time points and quenched in acetonitrile containing diclofenac (internal standard). The samples were then centrifuged and the supernatant was analyzed by UHPLC using a Kinetex 2.6 μm C18 (Phenomenex) column and a Shimadzu Nexera UHPLC. Verapamil was used as a positive control.

### Liver microsome (LM) stability assay

Compound at 1 μM (0.5% final DMSO concentration) was incubated with a 0.5 mg/mL CD-1 mouse (Sigma), SD rat (Life Technologies), or human liver microsome (Life Technologies) in the presence of 1 mM NADPH at 37°C for up to one hour. Aliquots were taken out at different time points and quenched in acetonitrile containing diclofenac (internal standard). The samples were then centrifuged and the supernatant was analyzed by LC-MS using a Luna Omega 1.6 μm Polar C18 (Phenomenex) column, a Shimadzu Nexera UHPLC, and a Sciex QTrap 5500 mass spectrometer. Verapamil was used as a positive control, and warfarin was used as a negative control.

### Pharmacokinetics (PK) in CD-1 mice

PK studies were performed in male CD-1 mice between 18 and 25 g in weight. The test compound was injected intravenously (i.v.) into CD-1 mice at 0.5 mg/kg in formulation containing 5% DMA (dimethyl acetamide) 15% Solutol HS15 and 80% water. Animals were euthanized at predefined time points by CO_2_ inhalation and blood was collected in K2-EDTA vacutainers by cardiac puncture. The vials were then centrifuged to collect plasma. The plasma samples were extracted using acetonitrile and then analyzed by LC-MS to determine the concentrations of Compound 1 and Compound 3. LC-MS was performed using a Luna Omega 1.6 μm Polar C18 (Phenomenex) column, a Shimadzu Nexera UHPLC, and a Sciex QTrap 5500 mass spectrometer. The PK parameters were calculated by non-compartmental analysis using Phoenix WinNonlin (Certara).

## Results

### Structure, thrombin inhibition, and selectivity

The structure of Compound 1 is shown in Fig 1, left, and IC_50_s for the inhibition of thrombin activity against a chromogenic peptide substrate are shown in Table 2.

**Table 2:** Enzyme inhibition IC_50_s for thrombin and other serine proteases.

IC_50_ (μM)	Argatroban	Dabigatran	Compound 1
**Thrombin**	0.22 ± 0.10	0.03 ± 0.02	0.06 ± 0.03
**Factor VIIa**	> 50	40.7 ± 13.3	13.8 ± 2.8
**Factor IXa**	> 50	> 50	> 50
**Factor Xa**	> 50	5.8 ± 2.9	8.3 ± 3.4
**Factor XIa**	> 50	4.2 ± 1.5	2.8 ± 0.2
**Factor XIIa**	> 50	> 50	> 50
**aPC**	> 50	41.3 ± 7.6	> 50
**Plasmin**	> 50	8.4 ± 2.6	0.7 ± 0.15
**Trypsin**	> 50	1.2 ± 0.1	31.6 ± 3.1

The assays were performed as described in the Materials and methods section.

When incubated with thrombin for 10 min, the IC_50_ against thrombin is 60 nM for Compound 1, 220 nM for argatroban, and 30 nM for dabigatran. The IC_50_ values for argatroban and dabigatran are consistent with the published apparent inhibition constants, K_*i*_, for these substrate-competitive direct thrombin inhibitors (DTIs), see for instance Lee [5] and Deftereos [30]. The IC_50_ for Compound 1 suggests that it is a potent inhibitor of thrombin with potency comparable to known DTIs. Furthermore, Compound 1 displayed similar potency against clot-bound thrombin with an IC_50_ of 100 nM.

The specificity of Compound 1 was examined by testing the extent to which the compound inhibits other serine proteases. The IC_50_s for argatroban, dabigatran, and Compound 1 against a panel of related serine proteases, including those involved in the coagulation cascade are shown in Table 2. Argatroban, dabigatran, and Compound 1 are all potent against thrombin, with argatroban being the most selective against the panel of related serine proteases.

Compound 1 showed >50x selectivity against all tested proteases except for plasmin and FXIa, against which it had >11x and >45x selectivity, respectively. These results suggest that Compound 1 has specificity comparable to dabigatran for the inhibition of thrombin.

Note that for a time-dependent covalent inhibitor such as Compound 1 (see”Thrombin inhibition kinetics” below), the IC_50_ can depend on both preincubation time and reagent concentrations. In this case, the ratio of the kinetic parameters *k*_inact_/*K*_I_, with *k*_Inact_ the rate of inactivation and *K*_i_ the apparent inhibition constant, can serve as a more reliable metric for the potency of the inhibitor. This analysis was performed for the enzymes against which Compound 1 showed an IC_50_ below 50 μM and is described in section”Selectivity against other serine proteases” below. Our findings support the conclusion that Compound 1 has high specificity for the inhibition of thrombin over other serine proteases.

### Compound 1 is a covalent inhibitor

**Fig 2:**
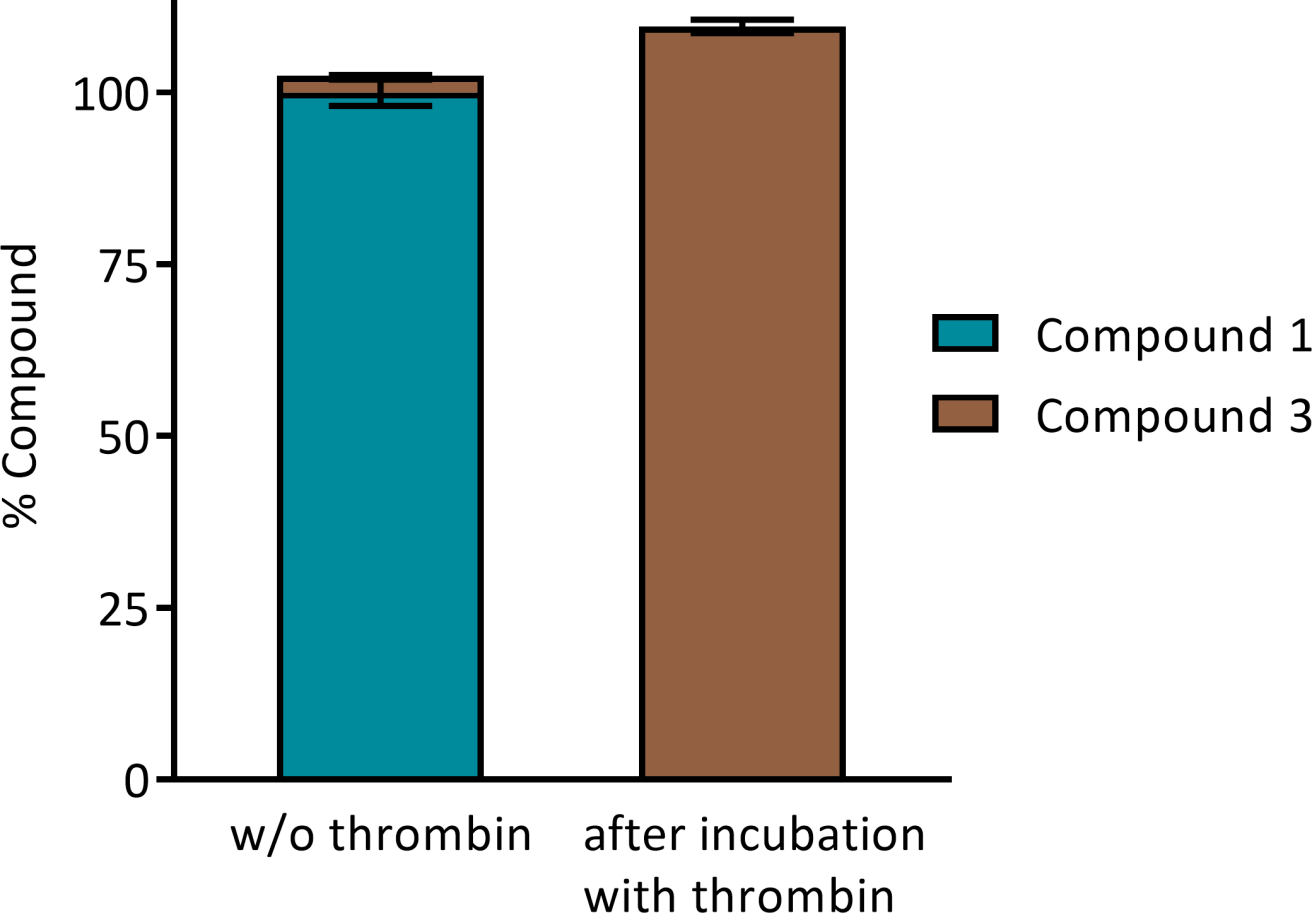
Interaction of Compound 1 with thrombin. Compound 1 at 1 μM was incubated with excess thrombin at 5 μM at room temperature for 20 min. The reaction was then quenched and monitored by LC-MS for Compound 1 and Compound 3. Note that quantitation levels were normalized to Compound 1 without thrombin. Stochiometric conversion of Compound 1 to Compound 3 was observed.

As discussed in section “Thrombin inhibition kinetics” below, Compound 1 is a time- and concentration-dependent thrombin inhibitor. Incubation of Compound 1 with thrombin for 20 min resulted in the consumption of the parent Compound 1 and the formation of a new species, which was identified using LC-MS/MS as a fragment of the parent (Compound 3, see Fig 1, right). The amount of Compound 3 formed was stoichiometric with the loss of Compound 1, as seen in Fig 2.

Incubation of Compound 1 with thrombin for a longer period (overnight) resulted in the release of 2-methoxybenzoic acid as detected by LC-MS in addition to the formation of Compound 3. A control sample with Compound 1 in buffer in the absence of thrombin is stable over the entire course of the experiment. Together with the crystallography experiments presented in the following, these findings strongly suggest that the 2-methoxybenzoyl group was transferred from Compound 1 to thrombin, resulting in deacylated Compound 3 and acylated thrombin.

Note that Compound 3 shows virtually no inhibitory activity against thrombin (IC_50_ > 100 μM).

#### Binding of dansyl Compound 2 to thrombin

To examine the binding mechanism of Compound 1, a close analog labeled with a dansyl group (Compound 2, shown in Fig 1, middle) was used. Compound 2 inhibits thrombin with potency similar to Compound 1, with an IC_50_ of 80 nM.

The dansyl group absorbs light in the spectral region where tryptophan residues (nine tryptophans in thrombin, all close to the active site) typically emit, i.e., 330–345 nm. Because of this spectral overlap, after irradiation of the protein sample at 280 nm, energy may be transferred through a non-radiative process from the tryptophan residues in the protein (donor) to the dansyl group of the compound (acceptor) resulting in a significant decrease of intrinsic fluorescence from the donor. The energy transfer is distance-dependent and the reported Förster radius, R_0_, for the tryptophan-dansyl pair is approximately 21 Å. Because most tryptophan residues in thrombin are located less than 15 Å away from the catalytic S195, significant fluorescence resonance energy transfer is expected if Compound 2 interacts with any of the tryptophan residues present in the active site.

**Fig 3:**
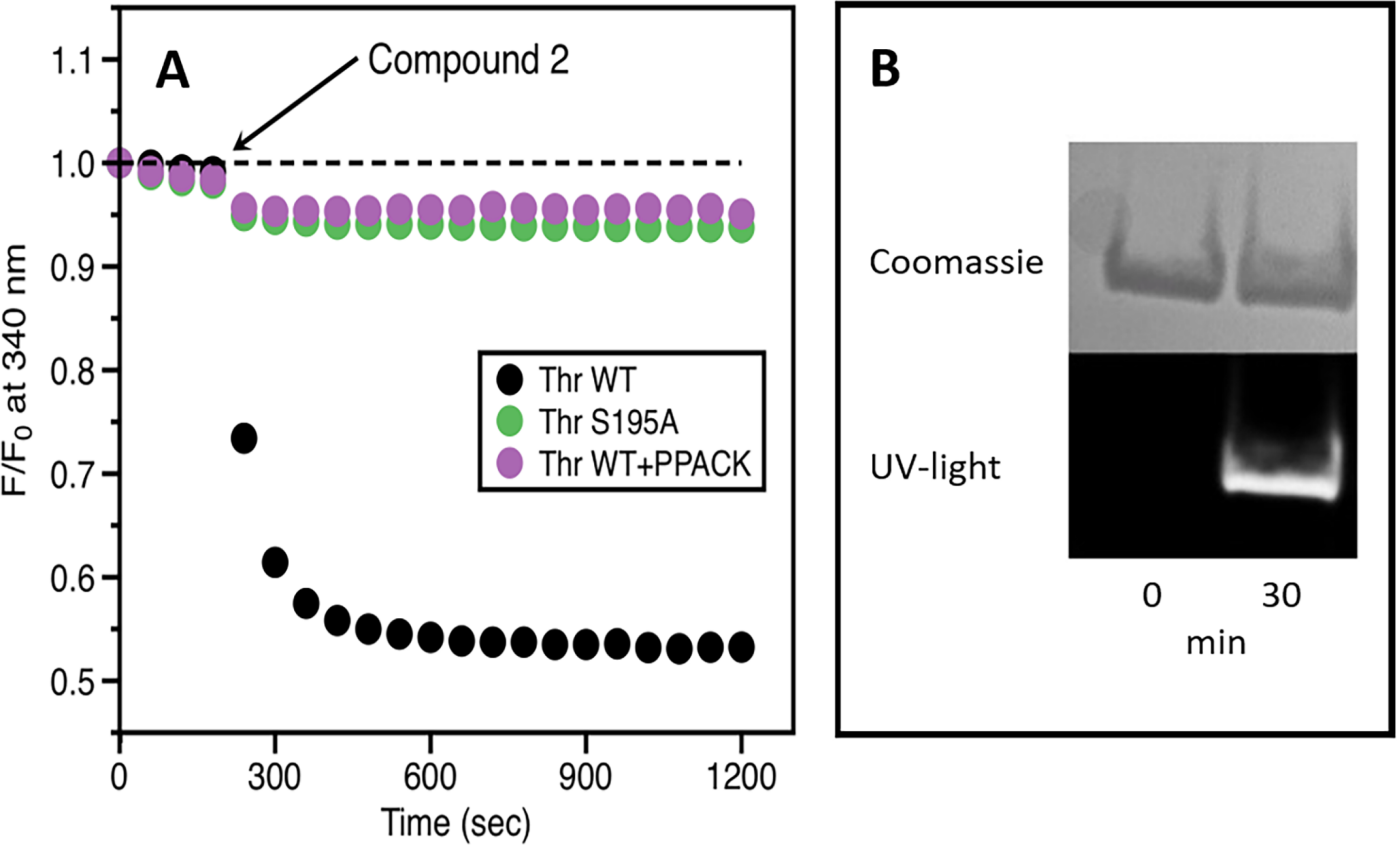
*Panel A*: Fluorescence intensity vs time for the incubation of Compound 2 with thrombin, thrombin mutant S195A, and thrombin with PPACK. After 3 min (arrow), the dansyl-labeled thrombin inhibitor Compound 2 was added to a solution of thrombin WT (black), thrombin preincubated with an excess of the irreversible active site inhibitor PPACK (magenta), or the active site mutant thrombin S195A (green) and incubated at room temperature for 2 h. The samples were monitored by fluorescence (excitation 280 nm, emission 340 nm). The observed differences in fluorescence quenching suggest that Compound 1 targets S195. *Panel B*: Compound 2 incubated with WT thrombin was analyzed by SDS-PAGE. Two samples are shown at time 0 and after 30 min. In the Coomassie-stained image (top), the thrombin band is visible irrespective of compound incubation. In contrast, when viewed under UV light (bottom), the thrombin band fluoresces only after incubation, indicating the incorporation of (parts of) Compound 2 into the enzyme.

When Compound 2 was incubated with thrombin, a time-dependent change in the fluorescence intensity at 340 nm was observed, indicating energy transfer between the enzyme and the inhibitor (Fig 3, panel A, black). Preincubation of thrombin with the irreversible inhibitor PPACK (D-phenylalanyl-prolyl-arginyl chloromethyl ketone; 100 μM) resulted in a lack of quenching (Fig 3, panel A, magenta). PPACK is known to form irreversible covalent bonds with both S195 and H57 in the active site [31], suggesting that both inhibitors may target the catalytic residues H57 and/or S195. This hypothesis was tested with the thrombin mutant S195A, which is structurally similar to wild-type thrombin but devoid of catalytic activity. Addition of 1 μM of Compound 2 to thrombin S195A failed to produce any change in fluorescence (Fig 3, panel A, green), which supports the hypothesis that Compound 1 or a fragment of Compound 1 covalently binds to S195.

Further evidence that Compound 1 is a covalent inhibitor of thrombin comes from examining the product of the reaction between Compound 2 and thrombin by SDS-PAGE after incubation for 30 min (Fig 3, panel B). Detection by Coomassie staining showed the expected thrombin band irrespective of incubation. Upon UV illumination, the thrombin band in the Compound 2-treated sample fluoresces, indicating stable incorporation of the compound—or a part of it—into the enzyme.

#### Protein crystal structure

A crystallography structure determination (PDB 6CYM, https://www.rcsb.org/structure/6CYM, DOI: 10.2210/pdb6CYM/pdb) furthermore shows that the catalytic serine residues in the two thrombin molecules in the asymmetric unit have been modified and that a probable methoxybenzene moiety is bound just beside S195. A small extra electron-density peak close to S195 was found in the heavy chain of each thrombin unit.

**Fig 4:**
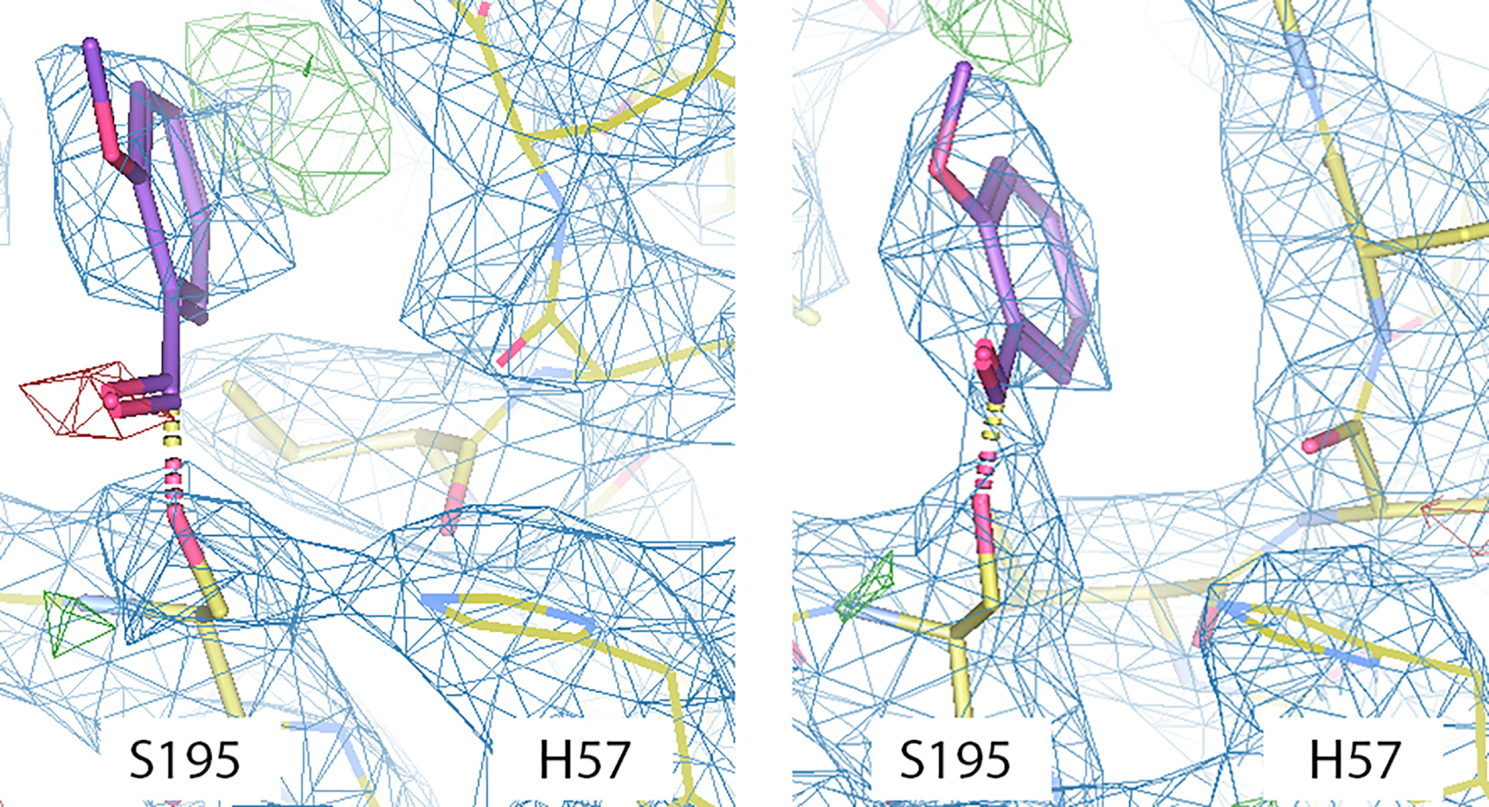
Structural model of the thrombin active site. The structural model of the thrombin active site is derived from X-ray crystallography of thrombin modified by Compound 1. The S195 in the active site required for thrombin enzymatic activity is modified by the 2-methoxybenzoyl group, rendering it inactive. In the monomer shown on the right, continuous electron density is found supporting the existence of a covalent linkage. In the monomer shown on the left, electronic density for the methoxyphenyl ring indicates blockage of the active site, but covalent linkage cannot be conclusively determined due to missing electron density (red).

In one monomer (Fig 4, right), continuous electron density is found supporting the existence of a covalent linkage. However, in the other molecule (Fig 4, left), electron density for the methoxyphenyl ring indicates blockage of the active site, but missing electron density (red) cannot conclusively determine covalent linkage. Given the totality of the data, we believe that the missing electron density is due to partial disorder of the carbonyl linkage, rather than the chemically unreasonable fragmentation of Compound 1 into a methoxyphenyl group.

The non-ideal quality of the structure is not an unexpected result as the crystallization process occurred over five days and deacylation was determined to take place with *k*_3_ = 1.4 x 10^−3^ min^-1^ (see section “Inhibition is reversible”), corresponding to a half-life of *t*_1/2_ ~ 8 h. Although off-rates are potentially slower in the crystal phase than in solution, due to the decreased water concentration in the crystal lattice, a significant fraction of acylserine sites are expected to be deacylated by the time of data acquisition. The apparent deacylation may be further mitigated by the excess of Compound 1 (0.2–0.5 mM) and the relatively rapid on-rate (*k*_inact_ < 0.1 sec^-1^) compared to *k*_3_. However, 100% acylation is not expected to be retained. Overall, the crystal structure is quite suggestive and we believe that in combination with the totality of our results (see below), there is indeed good evidence supporting covalent linkage.

**Fig 5:**
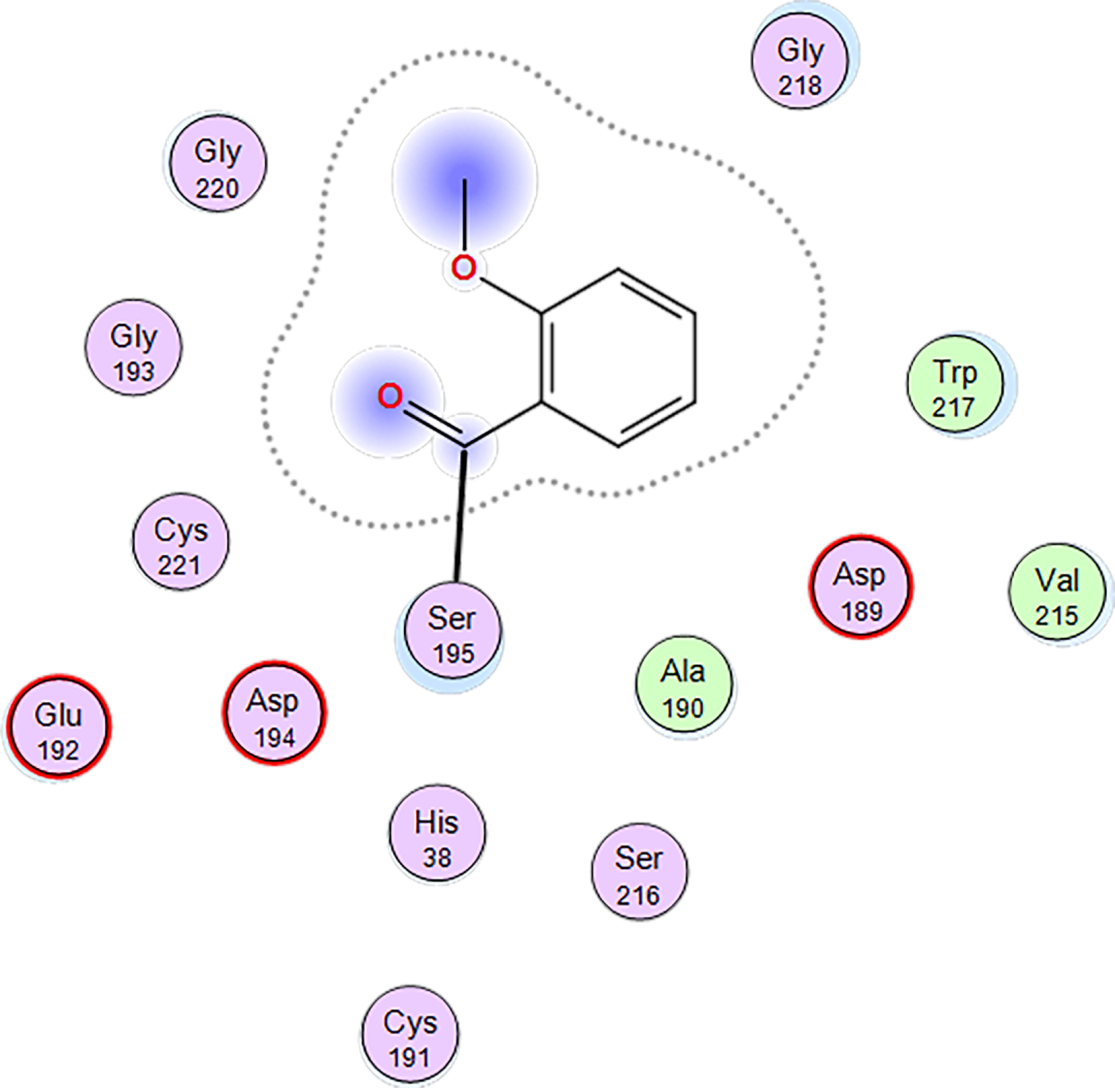
Interaction diagram for the thrombin active site modified by Compound 1. The illustration shows that no specific contacts between the 2-methoxybenzoyl group and thrombin are made.

Furthermore, due to the small size of the methoxybenzoyl group, the acylserine is likely disordered, which could also lead to the ambiguous densities observed. An interaction diagram (Fig 5) indicates that no specific contacts with thrombin are being made, including a lack of contact between the acylserine carbonyl and the oxyanion hole formed by G193 and S195.

### Thrombin inhibition kinetics

**Fig 6:**
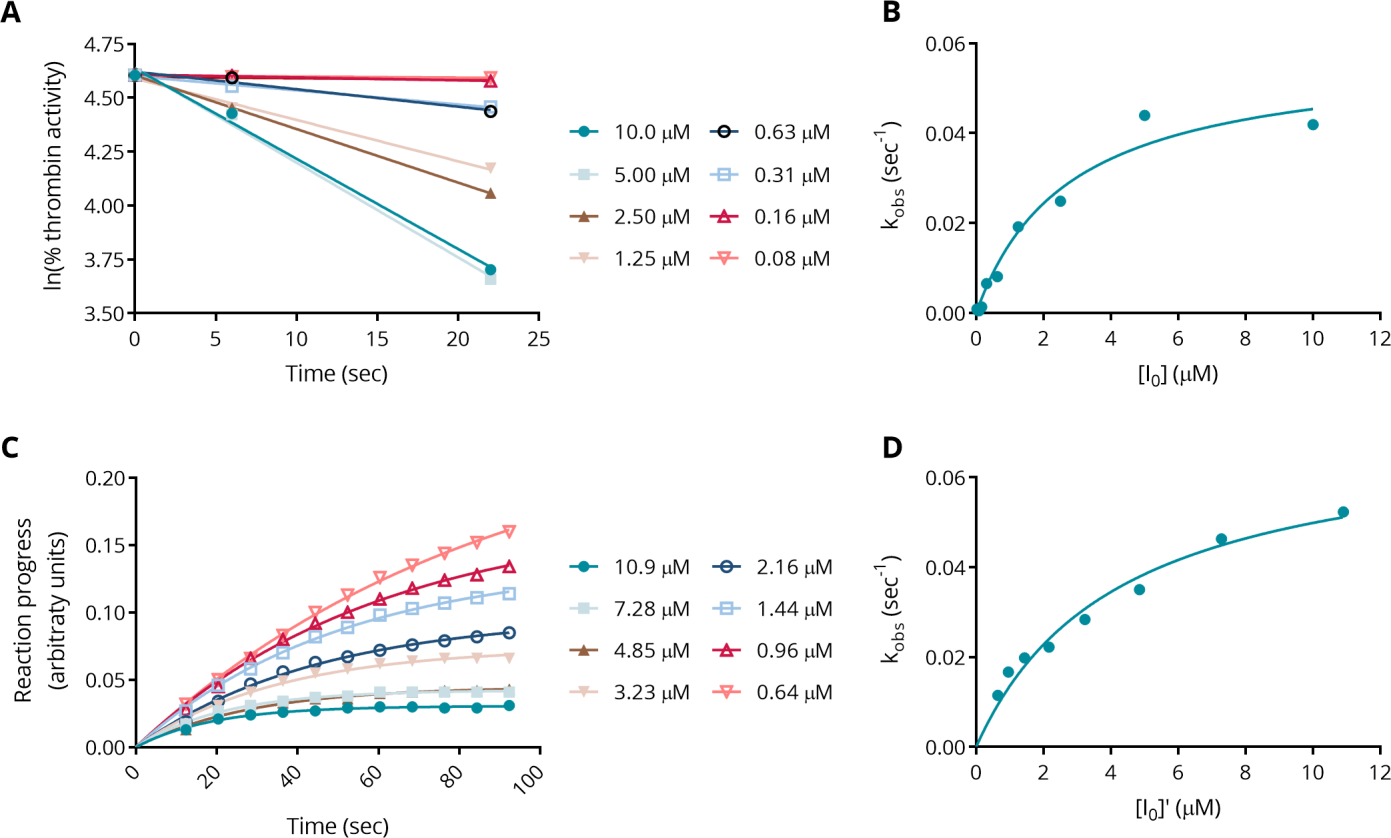
Kinetic characterization of Compound 1. *Panel A*: Remaining thrombin activity following preincubation with varying concentrations of Compound 1 for one representative experiment. *Panel B*: Plot of the unimolecular rate constant, *k*_*obs*_, determined from the slopes of the linear fits shown in panel A, as a function of initial inhibitor concentration. Shown is the fit to the Michaelis-Menten model. This method yields the kinetic parameters *K*_i_ = 3.2 ± 0.75 μM and *k*_inact_ = 0.08 ± 0.04 sec^-1^. *Panel C*: Reaction progress curves for thrombin inhibition at varying concentrations of Compound 1 for one representative experiment. Note that the legend shows modified inhibitor concentration. *Panel D*: Plot of the unimolecular rate constant *k*_*obs*_ determined from the time constants from the one-phase exponential association fits in panel C, as a function of modified inhibitor concentration. Shown is the fit to the Michaelis-Menten model. This method yields the kinetic parameters *K*_i_ = 2.1 ± 1.8 μM and *k*_*i*nact_ = 0.06 ± 0.02 sec^-1^.

Thrombin inhibition kinetics were studied as described in “Preincubation kinetics experiments” above. From the natural logarithm of % thrombin activity plotted against preincubation time (see Fig 6, panels A and B), the rate of inactivation, *k*_inact_, and the apparent inhibition constant, *K*_i_, were determined. For Compound 1, *K*_i_ = 3.2 ± 0.75 μM and *k*_inact_ = 0.08 ± 0.04 sec^-1^, which corresponds to a half-life of inactivation of about 9 sec, indicating a fairly slow on-rate of inhibition.

These kinetic parameters were corroborated in a second assay format, in which thrombin inhibition was observed in real time in the presence of the substrate (see Fig 6, panels C and D, and section”Progress curve kinetics experiments” above). Fitting the reaction progress curves to an exponential association model resulted in the values *K*_i_ = 2.1 ± 1.8 μM and *k*_inact_ = 0.06 ± 0.02 sec^-1^, consistent with those observed in the preincubation experiments.

### Inhibition is reversible

Moreover, the reversibility of the inhibition by Compound 1 was tested. Inhibition of serine proteases by acylation is known to be spontaneously reversible by hydrolysis of the acyl group [32].

**Fig 7:**
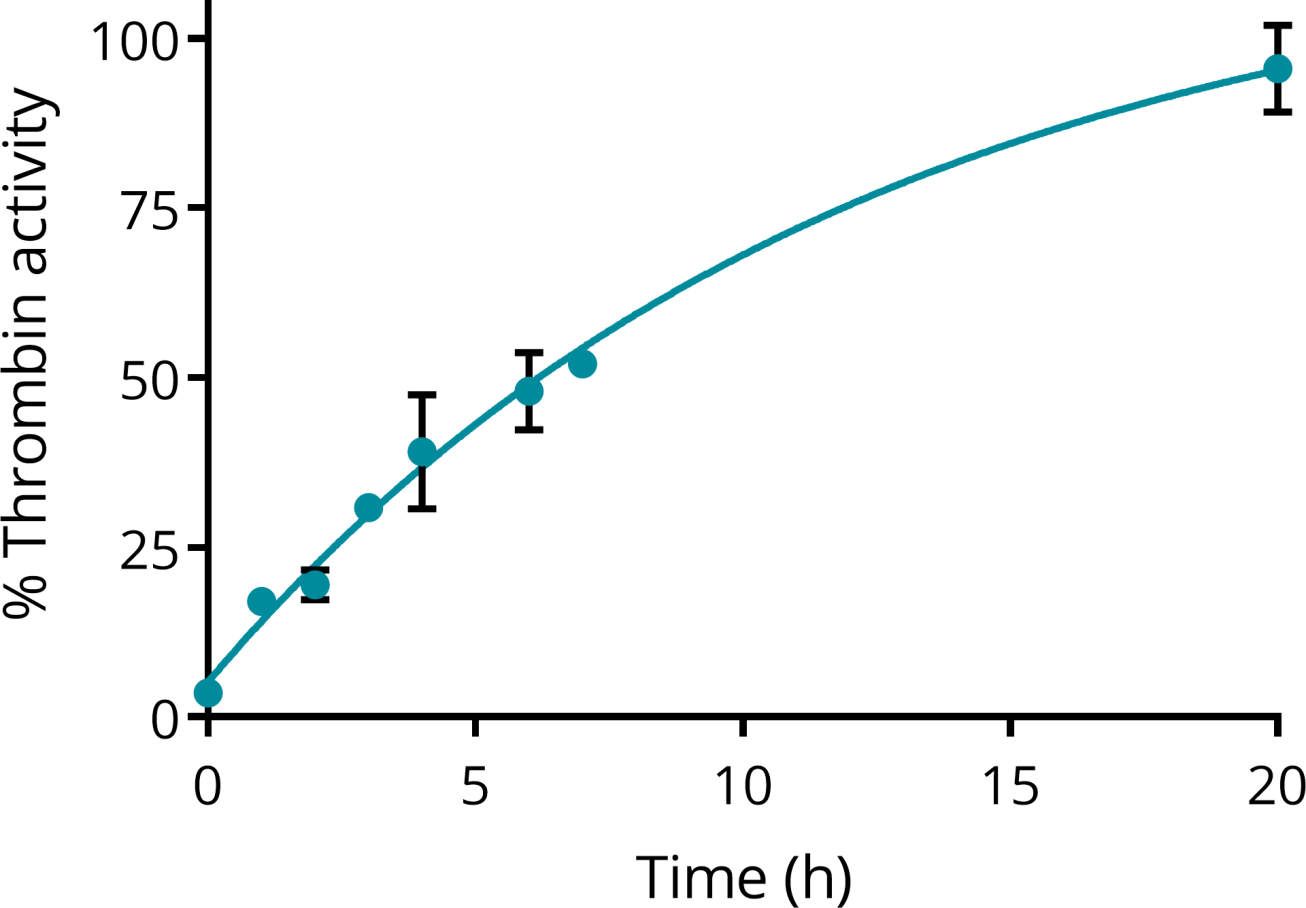
Spontaneous recovery of thrombin activity. % Thrombin activity was recorded as a function of time after inhibition by Compound 1 and removal of excess Compound 1. Fitting an exponential yielded a rate constant *k*_3_ = 1.4 x 10^−3^ min^-1^, which corresponds to a half-life of about 8 h.

The slow reversal of inhibition by Compound 1 was confirmed using a kinetic assay. Thrombin was first fully inhibited by an excess of Compound 1, after which the excess was rapidly removed and aliquots of the sample were assayed for thrombin activity, showing reversal of the inhibition (Fig 7). The data were fitted to an exponential, yielding a rate constant *k*_3_ = 1.4 x 10^−3^ min^-1^, which corresponds to a half-life of about 8 h.

### Selectivity against other serine proteases

The specificity of Compound 1 was examined by testing the extent to which the compound inhibits other serine proteases. The IC_50_ values for argatroban, dabigatran, and Compound 1 against a panel of related serine proteases, many of which are involved in the coagulation cascade, are shown in Table 2.

Since Compound 1 is a time-dependent covalent inhibitor, the second-order rate constant *k*_inact_/*K*_i_ was determined for the proteins with IC_50_ below 50 μM. The kinetic parameters, see Table 3, were derived using the progress curve method introduced in section”Progress curve kinetics experiments” above. In some instances, such as FVIIa and trypsin, saturation kinetics were not observed and *k*_inact_ and *K*_i_ could not be obtained explicitly. For these enzymes, the slope of a linear fit of the plot of *k*_obs_ versus [I]’_0_ was used to determine the ratio *k*_inact_/*K*_I_.

**Table 3:** Kinetic parameters for the inhibition of serine proteases.

	k_inact_/K_I_ (sec^-1^M^-1^)	k_inact_ (sec^-1^)	K_I_ (μM)
**Thrombin** (preincubation kinetics)	25,000	0.08 ± 0.04	3.2 ± 0.75
**Thrombin** (progress curve kinetics)	28,571	0.06 ± 0.02	2.1 ± 1.8
**Factor VIIa**	50 ^a^	—	—
**Factor Xa**	319	0.03 ± 0.03	94 ± 57
**Factor XIa**	109	0.0006 ± 0.0003	5.5 ± 4
**Plasmin**	5000	0.004 ± 0.002	0.8 ± 0.6
**Trypsin**	9^a^	—	—

^a^ Saturation for the k_obs_ vs [I]’ plot for these enzymes was not observed. k_inact_/K_i_ was derived from the slope of the plot instead.

The rate constant *k*_inact_/*K*_i_ indicates that Compound 1 is 5x more selective for thrombin than plasmin, and 70x more selective for thrombin than all other enzymes, see Table 3. Interestingly, the inhibition constant is lower against plasmin (*K*_i_ = 0.8 μM) than against thrombin (*K*_i_ = 2.1–3.2 μM), but the inactivation rate constant is also significantly lower for plasmin (*k*_inact_ = 0.004 sec^-1^) compared to thrombin (*k*_inact_ = 0.06–0.08 sec^-1^), making Compound 1 a more potent thrombin inhibitor. In comparison with the other enzymes, it appears that Compound 1 has a larger binding constant and lower inactivation rate compared to thrombin.

These results confirm our findings in section”Structure, thrombin inhibition, and selectivity” that Compound 1 has specificity for the inhibition of thrombin over the other serine proteases.

### Thrombin activity with endogenous substrates

Thrombin plays a pivotal role in the blood coagulation process. It is responsible for the cleavage of fibrinogen, which generates insoluble fibrin, making up the bulk of the clot. Thrombin also activates a number of procoagulant serine proteases such as FV, FVIII, FXI, and FXIII. When bound to its cofactor thrombomodulin, thrombin becomes an efficient activator of protein C, which acts as an anticoagulant and down-regulates thrombin. Another function of the thrombin/thrombomodulin complex is to activate the thrombin-activatable fibrinolysis inhibitor (TAFI), another procoagulant.

Hemostasis is a balance of the various procoagulant and anticoagulant activities of thrombin. We have examined the potencies of Compound 1 to inhibit some of these activities of thrombin and compared them to data for the known DTIs.

Table 4 summarizes the IC_50_ values for argatroban, dabigatran, and Compound 1 against endogenous substrates. The DTIs have nanomolar potency against protein C and TAFI activation by the thrombin/thrombomodulin complex with Compound 1 showing similar IC_50_s. Argatroban and dabigatran have potencies of 0.34 μM and 0.09 μM against fibrinogen cleavage, respectively, while Compound 1 has an IC_50_ of 4.2 μM.

**Table 4:** IC_50_s for the inhibition of endogenous substrates.

IC_50_ (μM)	Argatroban	Dabigatran	Compound 1
**Fibrinogen cleavage**	0.34 ± 0.11	0.09 ± 0.05	4.20 ± 0.17
**Activation of protein C**	0.007 ± 0.002	0.005 ± 0.004	0.014 ± 0.004
**Activation of TAFI**	0.19 ± 0.04	0.22 ± 0.04	0.340 ± 0.050

### Thrombin generation assay

The thrombin generation assay (TGA) is a plasma-based assay that measures the ability of the coagulation cascade to generate thrombin under conditions that resemble the physiological conditions involved in blood clotting. The TGA proceeds in two phases: an initial lag (initiation phase) followed by clotting and the rapid generation of thrombin (propagation phase). The results of the assay are expressed as a thrombogram, a thrombin generation curve in which the thrombin concentration (derived from a calibration curve) is plotted against time. The most relevant parameters derived from the TGA are the time to peak (T_max_), the peak height (C_max_), and the area under the thrombogram curve (endogenous thrombin potential, ETP) [33,34].

**Fig 8:**
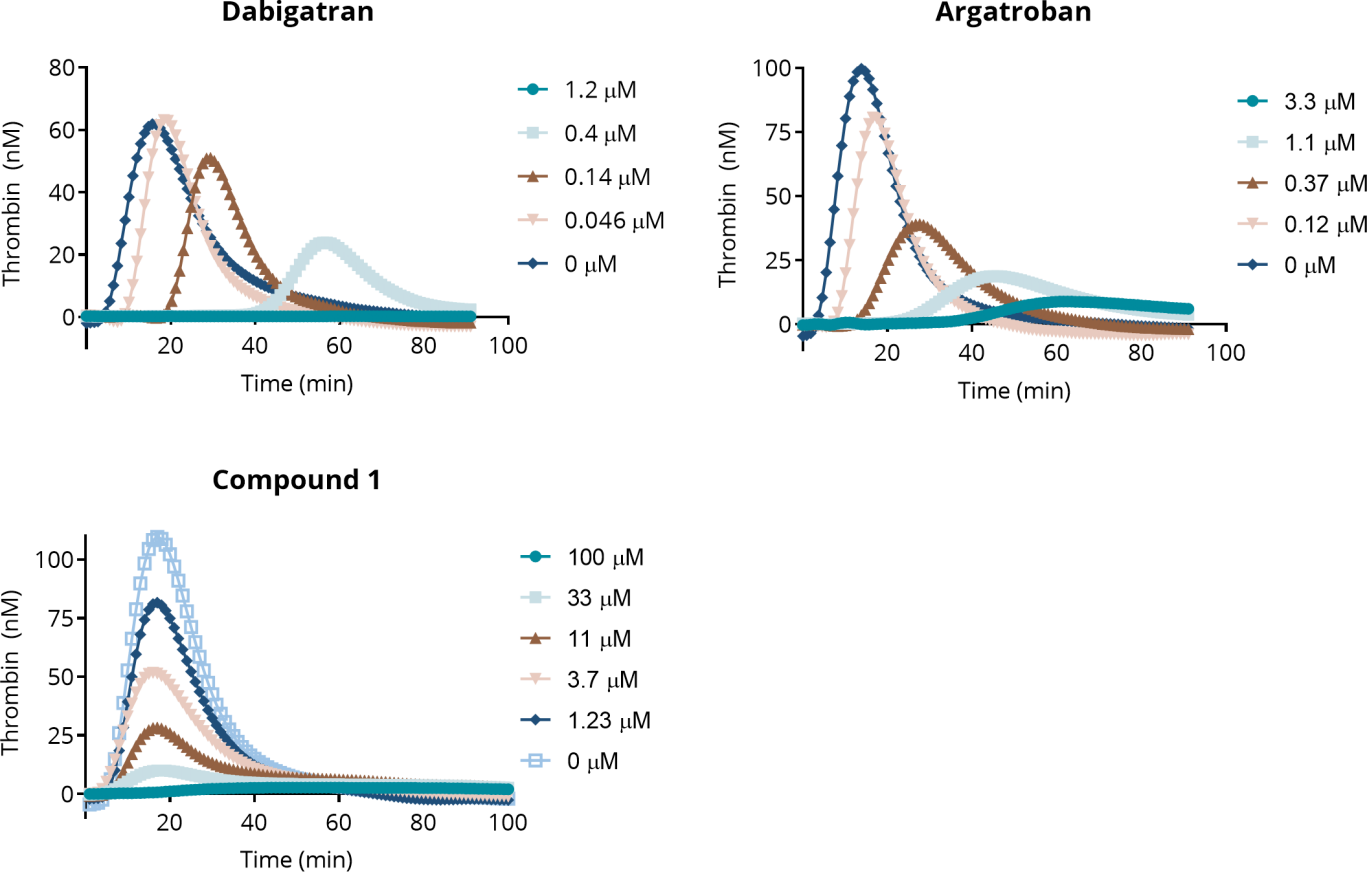
Thrombograms from the thrombin generation assay for argatroban, dabigatran, and Compound 1. The assays were performed as described in section “Thrombin generation assay (TGA)” above.

The TGA is responsive to many anticoagulant drugs, including various DTIs and FXa inhibitors. Thrombograms for argatroban, dabigatran, and Compound 1 are shown in Fig 8. The inhibition of thrombin generation by all three compounds is clearly reflected in the decreased peak amplitudes with increasing inhibitor concentration.

The measured ETP EC_50_ values are 0.34 μM for argatroban, 0.21 μM for dabigatran, and 4.1 μM for Compound 1 (see also Table 5).

**Table 5:** Thrombin generation assay parameters.

(μM)	Argatroban	Dabigatran	Compound 1	Compound 3
**ETP EC**_**50**_	0.34 ± 0.11	0.21 ± 0.04	4.1 ± 1.2	> 100
**C**_**max**_ **EC**_**50**_	0.27 ± 0.05	0.24 ± 0.08	3.65 ± 1.3	> 100
**T**_**max**_ **EC**_**2x**_	0.45 ± 0.07	0.06 ± 0.04	49 ± 15	> 100

Endogenous thrombin potential EC_50_ (ETP EC_50_), peak height EC_50_ (C_max_ EC_50_), and concentration necessary to shift the time to peak 2-fold (T_max_ EC_2x_) were recorded.

A significant difference between the thrombograms for Compound 1 and the known DTIs is observed in the lag time before the onset of thrombin growth. For argatroban and dabigatran, the lag times increase as the amplitude of the thrombin peak decreases, which is reflected in their low T_max_ EC_2x_ values (concentration required to shift the T_max_ 2-fold) shown in Table 5. The T_max_ EC_2x_ values for argatroban and dabigatran are 0.45 μM and 0.06 μM, respectively, i.e., similar to their ETP EC_50_ values. In contrast, the T_max_ EC_2x_ for Compound 1 is 49 μM and there was little or no increase in lag time up to concentrations corresponding to almost complete thrombin inhibition.

Note also that the product of the interaction of Compound 1 with thrombin, Compound 3, does not inhibit thrombin (ETP EC_50_ > 100 μM) in the TGA, see Table 5.

### Clotting assays

Compound 1 was tested in the clotting assays PT and aPTT as described in the Materials and methods section. These assays are routinely used to measure the coagulation status of human blood by measuring the time to clot upon initiation of the coagulation cascade with an excess of either tissue factor or particulated silica, mixed with phospholipids and calcium. These assays simulate the extrinsic and intrinsic coagulation pathways [35], respectively. They can be used to determine the concentration of compound required to shift the clotting time by 2-fold (CT_2x_). Compound 1 was found to be inactive in both these assays with CT_2x_ > 100 μM. The DTI argatroban, which was used as a positive control, showed a CT_2x_ of 0.60 ± 0.14 μM and 0.27 ± 0.15 μM in the PT and aPTT assays, respectively.

### Pharmacokinetics

Selected *in vitro* DMPK properties of Compound 1, such as its plasma and LM stability, were also tested in a number of different species. As shown in Table 6, the stability (half-life) of Compound 1 is very low in both mouse and rat plasma and in the presence of LMs, predicting poor PK for the compound in these species. In contrast, Compound 1 showed higher stability in human plasma and LM, indicating potential for good exposure in humans.

**Table 6:** *In vitro* plasma and LM stability of Compound 1.

*t*_½_ (min)	CD-1 mouse	SD rat	Human
**Plasma stability**	14 ± 1	25 ± 0	89 ± 9
**LM stability**	20 ± 2	8.0 ± 1.6	110 ± 0

*In vitro* plasma and LM stability for Compound 1 were tested in mouse, rat, and human. Results are given as mean ± standard deviation, *n* = 2.

Indeed, a PK study of Compound 1 in mice revealed that, as predicted by the *in vitro* stability, the compound has high i.v. clearance (t_½_) and very low exposure (AUC, see Table 7). The plasma samples were also analyzed for Compound 3, the deacylated form of Compound 1. While Compound 3 was detected in the plasma, its exposure was even lower than that of Compound 1. AUC of Compound 3 was about half the AUC of Compound 1 (Table 7).

**Table 7:** PK parameters after i.v. injection of Compound 1 in CD-1 mice.

Intravenous PK parameters	Compound 1	Compound 3
**t**_**½**_ (h)	1.9	N.D.^a^
**C**_**0**_ (ng/mL)	260	Not applicable
**AUC** (h∙ng/mL)	88	45.5
**CL** (mL/kg/h)	11000	N.D.^a^
**V**_**d**_ (mL/kg)	30000	N.D.^a^

Half-life (t_1/2_), initial concentration (C_0_), area under the curve (AUC), clearance (CL), and volume of distribution (V_d_) were recorded after i.v. injection of Compound 1 in CD-1 mice. For PK graph see S1 Fig.

^a^ Not determined due to insufficient number of data points above the lower limit of quantitation.

## Discussion

We have developed a class of direct thrombin inhibitors (VE-DTIs) that are potent and selective against other related proteases. Here, we have focused on an exemplary member of this class, Compound 1, to describe the mechanism of action of this proprietary DTI.

In a thrombin enzyme assay, Compound 1 displayed activity comparable to dabigatran. Furthermore, Compound 1 showed much weaker inhibitory activity against several other serine proteases, many of which are part of the coagulation cascade.

Our experiments show that the activity of Compound 1 is mediated by the whole molecule. In particular, the activity of Compound 1 requires the presence of the acylpyrazole group, as the deacylated Compound 3 has virtually no inhibitory activity against thrombin up to 100 μM. This observation illustrates the requirement for a complete set of structural features to achieve potency.

We have conducted a number of experiments that support a proposed mechanism of action where Compound 1 covalently modifies the active site S195 of thrombin. The inhibition is time dependent with *k*_inact_ < 0.1 sec^-1^, indicating a slow on-rate. A dansylated analog of Compound 1, Compound 2, produced a time-dependent change in tryptophan fluorescence, which was blocked if thrombin was preincubated with the irreversible active site inhibitor PPACK or the active site mutant S195A. Gel electrophoresis studies under denaturing conditions showed stable incorporation of the dansyl moiety upon incubation of thrombin with the dansyl-containing Compound 2. Together, these results confirm that Compound 1 targets the active site S195 of thrombin.

Incubation of Compound 1 with thrombin leads to complete conversion of Compound 1 to the deacylated fragment Compound 3. The crystal structure of thrombin seeded with Compound 1 showed electron density consistent with the 2-methoxybenzoyl group transferred to S195. Even though the crystal structure by itself is not fully conclusive, the combination of our findings strongly suggest that it is indeed the 2-methoxybenzoyl group that is transferred.

**Fig 9:**
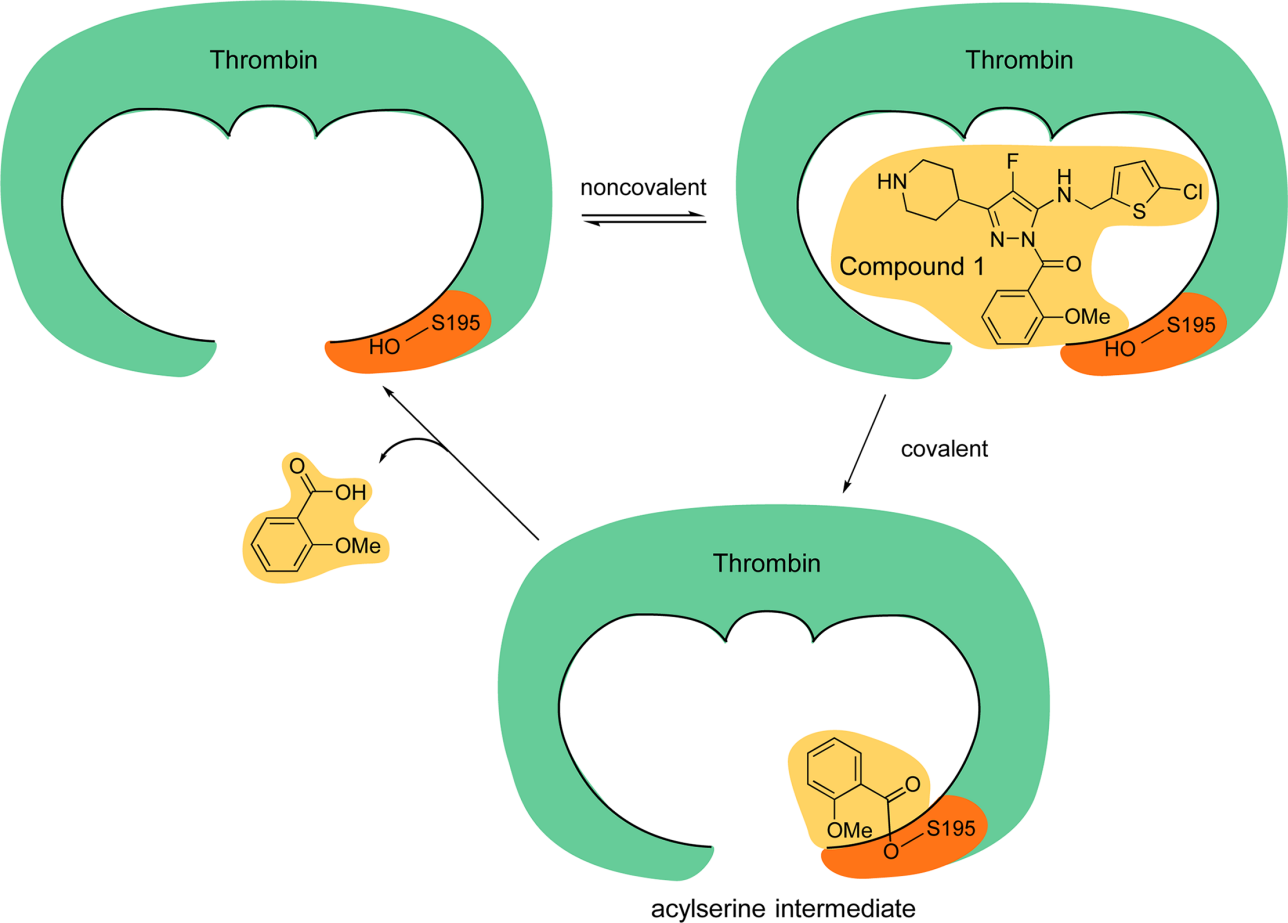
Proposed mechanism of action of Compound 1. Compound 1, an exemplary VE-DTI, binds to thrombin and orients to react with the active site S195, which results in its acylation. The modified form of thrombin is inactive until deacylation of S195.

We have also shown that the inhibition is reversible, as seen by the spontaneous, slow recovery of thrombin activity over time. Further validation of the reversibility of the inhibition is provided by the release of the acyl group 2-methoxybenzoic acid following incubation of thrombin with Compound 1, as mentioned in section”Compound 1 is a covalent inhibitor” above. This is exactly the moiety that would be released as per our model described below and shown in Fig 9.

The proposed mechanism of action for Compound 1 is that initial binding of the compound to thrombin is followed by covalent modification of the catalytic S195 required for thrombin proteolytic activity by acyl transfer of the 2-methoxybenzoyl group. This is eventually followed by hydrolysis and regeneration of thrombin activity.

The key features of this mechanism are presented in Fig 9. Initially, Compound 1 approaches the active site of the protease in a reversible fashion. Next, the compound is cleaved by nucleophilic attack by the catalytic S195. The 2-methoxybenzoyl group is transferred from the inhibitor to the enzyme by acylation of the serine hydroxyl group. The deacylated fragment (Compound 3) leaves the active site. Finally, the 2-methoxybenzoyl group is released, presumably by hydrolysis, and the active enzyme is regenerated.

Compounds similar to Compound 1, which reversibly acylate serine proteases, have been previously described. For example, a 1,2,4-triazole derivative has been shown to covalently and reversibly acylate serine proteases such as human tissue kallikreins and matriptase [32]. Our data indicate that this mechanism of action leads to novel pharmacology, which makes Verseon’s reversible covalent direct thrombin inhibitors promising candidates for drug development.

The activities of Compound 1, dabigatran, and argatroban were tested against the physiological substrates of thrombin such as fibrinogen, protein C, and TAFI. These studies indicate that the VE-DTIs are effective nanomolar inhibitors against thrombin/thrombomodulin activities. Furthermore, Compound 1 is a single-digit micromolar inhibitor against the cleavage of fibrinogen by thrombin.

A difference between Compound 1 and the competitive, noncovalent inhibitors argatroban and dabigatran was observed in the timing of peak thrombin activity following initiation of the coagulation cascade in the TGA. The presence of the competitive, noncovalent inhibitors resulted in a dose-dependent increase in lag time before the onset of thrombin growth. This feature has also been observed with other DTIs and FXa inhibitors like apixaban [36]. In contrast, the lag time is almost unchanged at the ETP EC_50_ for Compound 1. Essentially, Compound 1 appears to be a weak inhibitor of thrombin initiation, but a potent inhibitor of thrombin propagation.

At the end of the initiation phase in the TGA, a low nanomolar amount of thrombin is formed [37, 38], which leads to initial clot formation. The absence of an increased lag time (high T_max_ EC_2x_) associated with Compound 1 may allow coagulation to start promptly without affecting the initiation phase of thrombin generation, while Compound 1 rapidly limits the extent of thrombin production during the propagation phase as seen by the potent ETP EC_50_.

The TGA profile of Compound 1 is characteristic of the VE-DTIs. We attribute this distinctive profile to the relatively slow deactivation time of thrombin compared to the rapid inactivation by noncovalent DTIs.

Compound 1 is a very weak inhibitor of coagulation in both the PT and aPTT assays. These clotting assays are often used as biomarkers for anticoagulants [39, 40]. Most of the known anticoagulants, including warfarin, heparin, and the DOACs, have potency in these assays at clinically relevant efficacious concentrations. It appears paradoxical that Compound 1, which inhibits purified thrombin at reasonable concentrations (IC_50_ = 0.06 μM) and shows potency in the TGA (ETP EC_50_ = 4.1 μM) is inactive in the clotting assays. However, these results are consistent with the observation that the duration of the lag phase of the TGA is weakly influenced by Compound 1: T_max_ EC_2x_ is high (around 50 μM), which allows an initial clot to form and for thrombin propagation to begin.

Unlike the TGA, which is believed to use physiologically relevant (about 1 pM) amounts of tissue factor, the PT and aPTT assays use very high non-physiological agonist concentrations. Moreover, the outputs of PT and aPTT reveal only the time at which clotting is macroscopically detected but provide no precise physical description of clot strength or composition. Our results are hence consistent with the weak observed activity in the PT or aPTT assays.

The mechanism of action of Compound 1 has been elucidated. Our studies, however, show that the compound does not have suitable PK properties for it to become a viable candidate for drug development.

We have developed a variety of compounds that have a similar mechanism of action to Compound 1 and tested their function in the assays described above. Many of these compounds have greater potency, selectivity, and more favorable pharmacokinetic properties suitable for preclinical development. These compounds are being optimized for potency and safety in various *in vivo* efficacy and bleeding models and will be reported in the future.

## Supporting information

S1 Dataset wwPDB validation report. (PDF)

S1 Fig Intravenous pharmacokinetics for Compound 1 measured in CD-1 mice. (TIF)
