# Supplementary figures and images for: Reversible covalent direct thrombin inhibitors
Source: PLoS One. 2018 Aug 2;13(8):e0201377. doi: 10.1371/journal.pone.0201377 (PMC6072017; doi:10.1371/journal.pone.0201377)

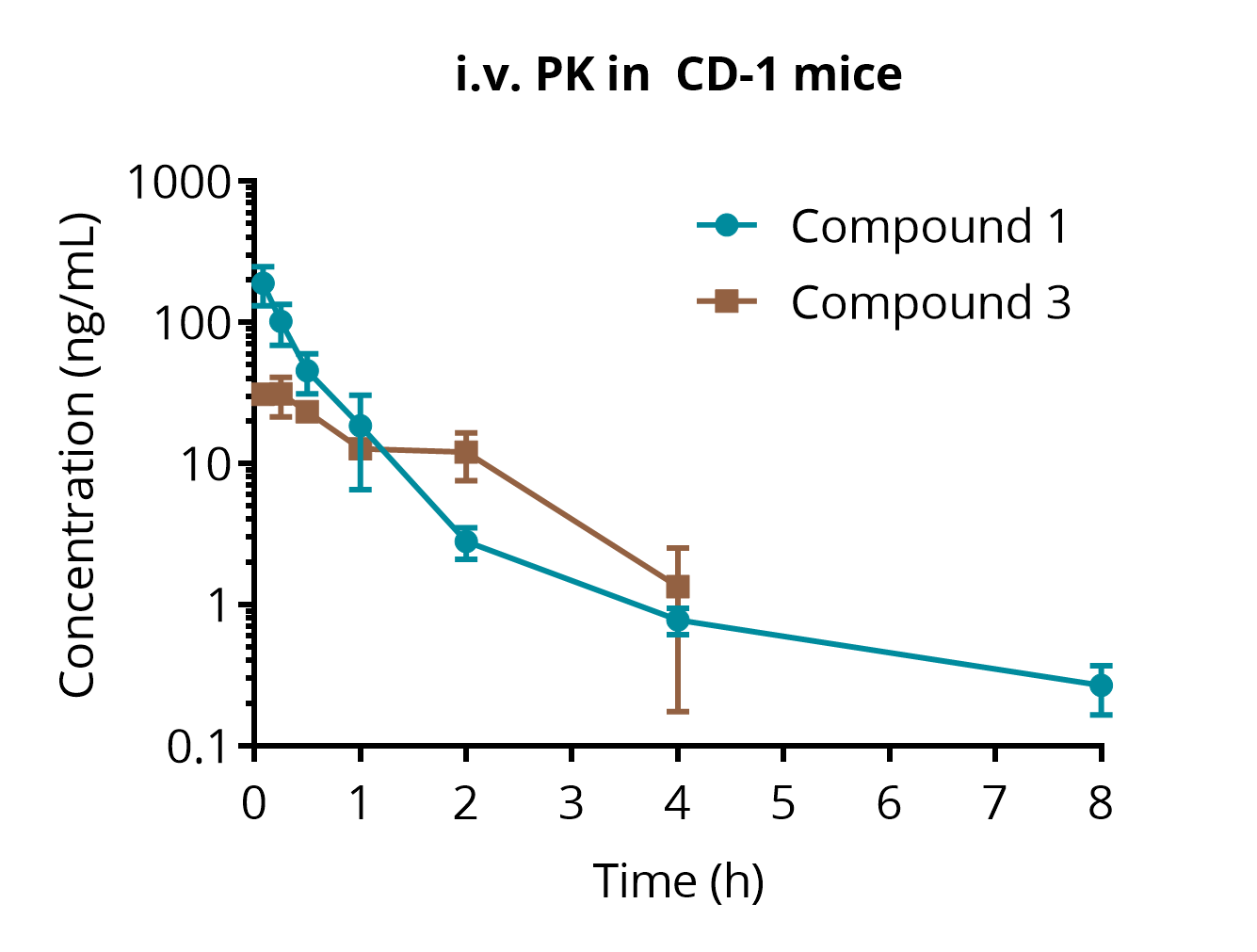

Supplement: S1 Fig — (TIF) [file pone.0201377.s002.tif]
